# Electronic Tongue Response to Chemicals in Orange Juice that Change Concentration in Relation to Harvest Maturity and Citrus Greening or Huanglongbing (HLB) Disease

**DOI:** 10.3390/s151229787

**Published:** 2015-12-02

**Authors:** Smita Raithore, Jinhe Bai, Anne Plotto, John Manthey, Mike Irey, Elizabeth Baldwin

**Affiliations:** 1 USDA-ARS, U.S. Horticultural Research Laboratory, 2001 South Rock Road, Fort Pierce, FL 34945, USA; smitare@gmail.com (S.R.); jinhe.bai@ars.usda.gov (J.B.); anne.plotto@ars.usda.gov (A.P.); john.manthey@ars.usda.gov (J.M.); 2 US Sugar Corp., 111 Ponce de Leon Ave, Clewiston, FL 33400, USA; mirey@ussugar.com

**Keywords:** sensor, quality, citrus, sugars, acids, limonoids, flavonoids, huanglongbing, harvest maturity, sensory

## Abstract

In an earlier study, an electronic tongue system (e-tongue) has been used to differentiate between orange juice made from healthy fruit and from fruit affected by the citrus greening or Huanglongbing (HLB) disease. This study investigated the reaction of an e-tongue system to the main chemicals in orange juice that impact flavor and health benefits and are also impacted by HLB. Orange juice was spiked with sucrose (0.2–5.0 g/100 mL), citric acid (0.1%–3.0% g/100 mL) and potassium chloride (0.1–3.0 g/100 mL) as well as the secondary metabolites nomilin (1–30 µg/mL), limonin (1–30 µg/mL), limonin glucoside (30–200 µg/mL), hesperidin (30–400 µg/mL) and hesperetin (30–400 µg/mL). Performance of Alpha MOS sensor sets #1 (pharmaceutical) and #5 (food) were compared for the same samples, with sensor set #1 generally giving better separation than sensor set #5 for sucrose, sensor set #5 giving better separation for nomilin and limonin, both sets being efficient at separating citric acid, potassium chloride, hesperitin and limonin glucoside, and neither set discriminating hesperidin efficiently. Orange juice made from fruit over the harvest season and from fruit harvested from healthy or HLB-affected trees were separated by harvest maturity, disease state and disease severity.

## 1. Introduction

Most of the oranges grown in Florida are used to make juice [1]. Consumer preference for juice largely depends on flavor quality, therefore, ensuring consistent flavor quality is of vital importance to juice processors. The epidemic of citrus greening disease, or Huanglongbing (HLB), however, is affecting the Florida citrus industry by ultimately killing the trees while imparting off-flavors to the fruit and processed juice [2]. One of the procedures that processors employ to maintain uniform flavor within batches is the blending of juices with different color and chemical composition resulting in a final product that meets the criteria for acceptable orange juice [3]. Therefore, it would be beneficial if juices from different batches, varieties or harvests could be screened quickly so that decisions could be made for blending. The important non-volatile flavor and mouthfeel drivers in orange juice are sugars, acids, salts, limonoids and flavonoids, respectively [4]. Especially important are the bitter limonoids, limonin and nomilin. These compounds are affected by HLB disease in that the disease can result in lower sugars, sometimes higher acids and higher levels of bitter limonoids and some flavonoids [4,5]. Although the amounts of potassium in HLB-affected and healthy juice were not found to be consistently different, HLB juice was found to be saltier or having more umami flavor [6] (Plotto, unpublished data). This can result in orange juice that is less sweet, more bitter, saltier and more astringent [2]. Generally, assessing a full flavor profile of orange juice includes determination of these compounds as well as conducting sensory panel evaluation. Conducting all these analyses is expensive, time consuming and sensory panels can be somewhat subjective. In the fast paced environment of a juice processing plant, a fast and objective method for sample classification would be useful. Recently, electronic tongue (e-tongue) technology has been used to assess the quality of a variety of foods [7,8,9,10,11].

The e-tongue is designed to roughly mimic the taste perception mechanism in humans. The human process involves interaction of taste molecules or stimuli with taste buds on the tongue generating multidimensional signals that are ultimately transmitted to the brain via neurons for final processing and pattern recognition with the final output that is taste perception [12]. In the case of the e-tongue, taste buds are replaced by an array of nonspecific sensors that respond to various chemicals in solution, as is found for food products, so that it is possible to discriminate complex solutions containing many ions [13]. This generates a complex set of multidimensional information which is then simplified and classified with the help of a computer with multivariate statistical software, such as principal component analysis (PCA, non-supervised) or linear discriminant analysis (LDA, supervised). In practice, analysis of samples by the e-tongue is very simple with the only criteria being solubilization in water, thus this technique is well suited for beverages. As such, various studies have reported the use of e-tongue technology to obtain data for taste attributes such as sourness, bitterness and astringency for beverages including beers, wines and teas [8,14,15]. While other analytical methodologies are targeted and classify samples based on a few chemicals, the e-tongue considers input from the whole sample so no extraction of samples is required. This also eliminates variability introduced when manipulating samples. Buellens *et al.* [9] showed that the e-tongue was able to discriminate between tomato cultivars containing varying amounts of sugars and acids, comparable to the more usual but more rigorous high pressure liquid chromatography (HPLC) analysis, suggesting that the e-tongue is an effective method for sample discrimination. Other studies have shown use of the e-tongue in distinguishing adulterated from unadulterated milk samples [10,16]. Furthermore, it has been shown in wine samples that it was not only possible to differentiate samples from different geographical areas, but also that the concentrations of multiple and diverse chemicals such as tartaric acid, malic acid, anthocyanins and total polyphenols could also be calculated by calibrating the electronic tongue [7].

Several studies have reported use of the electronic tongue to distinguish orange juice or drink products from different commercial brands with different chemical compositions [11,17,18]. To our knowledge there has been only one report of analysis of orange juice affected by HLB disease [19]. The authors reported that the e-tongue used was able to distinguish orange juice from fruit harvested from healthy trees compared to juice from fruit harvested from HLB-affected trees. More differences were observed for juice from HLB-affected fruit that were symptomatic for the disease (HLBs, small green and lopsided) than those that were asymptomatic for the disease (HLBa). It is still not known which compounds elicited sensor responses when differences between healthy and HLB-affected orange juice were observed.

The e-tongue could be a useful tool for orange juice processors for screening of juice made from HLB-affected fruits for quality control. Therefore, the objectives of this paper were to understand the response functioning of the etongue sensors by using the Alpha MOS α-ASTREE e-tongue system to segregate orange juice samples spiked with different concentrations of the chemicals important to flavor and health benefits of orange juice that are also impacted by HLB.

## 2. Materials and Methods

### 2.1. Chemicals

Sucrose was purchased from a local supermarket (Publix Super Markets, Fort Pierce, FL, USA), citric acid from SAFC Supply Solutions (St. Louis, MO, USA), potassium chloride from Sigma Aldrich (St. Louis, MO, USA), limonin and nomilin from 2A PharmaChem USA (Lisle, IL, USA) and ethanol (200 Proof-Absolute, Anhydrous ACS/UPS Grade) from Pharmco-AAPER (Brookfield, CT, USA). Hydrochloric acid, sodium chloride and monosodium glutamate used for assessing the performance and calibration of the electronic tongue were obtained from Alpha-MOS (Toulouse, France). Limonin glucoside, hesperetin and hesperidin were obtained from purified authentic standards.

### 2.2. Base Orange Juice

To understand which chemicals are being detected by the e-tongue sensors, a base orange juice sample was spiked with various chemicals known to change due to the presence of HLB disease. For this purpose, ten one U.S. gallon jugs of orange juice (Great Value 100% Orange juice, from concentrate, pasteurized) were purchased from a local supermarket (Wal-Mart Stores, Inc., Fort Pierce, FL, USA). To ensure sample homogeneity, juice from all the ten jugs were pooled together, shaken to mix, allocated into 1 L glass jars and stored at −20 °C freezer until further use. Prior to e-tongue analysis, juices were thawed at room temperature and centrifuged at 27,100 g at 4 °C for 20 min to precipitate the water insoluble materials out of the juice. The resulting supernatant was then filtered with four layers of cheesecloth to remove any particulates that could alter the performance of the e-tongue sensors. The sugar, acid, salt, limonoids and flavonoids described below were spiked into this juice at different levels and this was repeated three times for each spiked compound. For each chemical, an unspiked sample was also analyzed as a control.

### 2.3. Field Orange Juice

Hamlin oranges were harvested weekly from Southern Gardens Citrus Groves in South Florida over the harvest season (December through March, 2011–2012), and processed in the Southern Gardens Citrus processing plant. Fruit were harvested from healthy trees or trees affected by HLB disease, determined by qPCR analysis of leaves for the presumed pathogen, *Candidatus* *Liberibacter asiaticus* [20,21]. The fruit harvested from HLB-affected trees were separated into HLBs and HLBa before processing [2,4]. Sample processing for electronic tongue (sensor set #5) analysis was similar to the spiked juice samples described above, including centrifugation and filtration prior to analysis.

### 2.4. Sugar, Acid and Salt Spiking of Orange Juice

Base juice was individually spiked with various concentrations of sucrose, citric acid and potassium chloride. Concentrations for spiking were chosen such that the typical values found in orange juice were between the lowest and the highest levels used in this study [4]. Since all three of these compounds are soluble in water, spiked samples were prepared by adding the appropriate amounts of sucrose (0.2, 0.4, 0.6, 1, 2, 3 and 4 g/100 mL), citric acid (0.1, 0.2, 0.4, 0.6, 0.8, 1, 2 and 3 g/100 mL) and potassium chloride (0.1, 0.2, 0.3, 0.5, 1, 2 and 3 g/100 mL).

### 2.5. Secondary Metabolite Spiking of Orange Juice

For this study, the secondary metabolites chosen were nomilin, limonin, limonin glucoside, hesperidin, and hesperetin, the aglycone of hesperidin. In contrast to sucrose, citric acid and potassium chloride, these secondary metabolites, with the exception of limonin glucoside, did not dissolve in water and therefore had to be brought into solution using organic solvents first. Limonin and nomilin were brought into solution by placing in a round bottom flask containing 3 mL ethanol and heating at 45 °C at a constant rotation for 1 h. The solutions were then cooled to room temperature and added to the centrifuged base orange juice to make 1, 10 and 30 µg/mL limonin and nomilin solutions. Hesperidin and hesperetin were first dissolved in 2 mL dimethyl sulfoxide (Sigma Aldrich) and then added to the base juice to give 30, 50, 100, 200 and 400 µg/mL of each compound. Preliminary experiments showed that the addition of small amounts of ethanol (3 mL) and dimethyl sulfoxide (2 mL) to the base orange juice did not affect sensor responses. Spiked samples of limonin glucoside (30, 50, 100, 150 and 200 µg/mL) were prepared by dissolving the appropriate amounts in the base juice.

### 2.6. Electronic Tongue Analysis

The e-tongue system used for sample analysis was the Alpha MOS ASTREE II Liquid Analyzer (Alpha MOS America, Hanover, MD, USA) consisting of a 16 position auto-sampler, an array of sensors, a reference electrode (Ag/AgCl) and a chemometric software package. Two different sensor arrays each containing seven sensors were used in this study: sensor set #1 (ZZ, JE, BB, CA, GA, HA, JB) and sensor set #5 (SRS, GPS, STS, UMS, SPS, SWS, BRS). Sensor set#1 is designed for pharmaceutical applications and set #5 is designed for food and beverage applications where functionality for each sensor has been defined. Sensor SRS responds to sourness, GPS to metallic, STS to saltiness, UMS to umami, SPS to spiciness, SWS to sweetness and BRS to bitterness. Regardless of the type, these sensors are based on Chemical modified Field Effect Transistor (Chem FET) technology and coated with proprietary membranes that impart the non-specificity, low selectivity qualities to the sensors as well as cross-sensitivity to different components in solution (www.alpha-mos.com). Contact with dissolved compounds changes the charge density of the membrane surface and ion distribution near the surface of the membrane. This change in charge density is measured as the electric potential difference between each of the seven sensors and the reference electrode.

Prior to analyzing the juice samples, the electronic tongue sensors were conditioned (0.01 mol/L hydrochloric acid), calibrated (0.01 mol/L hydrochloric acid) and tested (diagnostic, 0.01 mol/L each of hydrochloric acid, sodium chloride and monosodium glutamate) for proper functioning and stability. Following successful tests, each juice sample was analyzed seven times for a period of 120 s. However since the sensor signals did not stabilize immediately, only the data from the last 20 s of the 4th, 5th, 6th and 7th run were used in the analysis. To avoid carry over effects, the sensors were rinsed in deionized water after each analysis. The raw data thus obtained was multivariate in nature and expressed as voltage *versus* time. Data analysis was done using the software package included in the electronic tongue system (Alpha MOs America). PCA was conducted on the raw data for pattern recognition and classification of samples for each chemical. The distance between two sample groups were measured by calculating the Euclidean distance between the centroids of the defined groups. This value is a practical means of evaluating the similarity between two sample groups. The greater the distance between the center of gravity for each group, the greater the differences between groups. The software package also allowed sensor optimization and the sensors that had discrimination power more than 0.5 were chosen when analyzing data.

### 2.7. Determination of the Chemical Composition of Base Juice

The concentrations of sugars and acids in the base juice were determined by using a previously described method in triplicates [4]. For secondary metabolites, 10 mL base juice along with 70 µL of 1.8 mg/mL mangiferin (Sigma Aldrich) as internal standard, was pipetted into a centrifuge bottle containing 30 mL methanol (Sigma Aldrich) and the mixture was shaken and heated at 55 °C for 15 min in a shake incubator to allow extraction of the analytes. The resulting solution was then centrifuged at 15,000 g at 5 °C for 20 min, the supernatant was transferred to a glass bottle and to the remaining pellet, an additional 10 mL deionized water and 30 mL methanol was added. The extraction was then repeated for the second time under the same conditions. The resulting supernatant was mixed with the supernatant obtained from the previous step and was concentrated by using a rotary evaporator to yield 2.5 mL extract. This extraction was repeated three times. The extract containing the secondary metabolites were then analyzed and quantified by using the method explained in Baldwin *et al.* [4].

### 2.8. Sensory Analysis

Hamlin juice was presented to a 12-member panel specifically trained (>50 h) for descriptive analysis of orange juice similar to Plotto *et al.* [2], with some modifications. Specific descriptors were chosen for the Hamlin healthy and HLB-affected juice. Samples were served as 50 mL juice in 110 mL cups (Solo Cups Company, Urbana, IL, USA). A healthy juice sample was served as a warm-up sample and rated in the same way as the test samples, and three juices, healthy, HLB symptomatic and HLB asymptomatic were presented in a randomized order. Panelists rated nine aroma and 20 flavor/taste/mouth feel descriptors using a 16-point intensity scale where 1 = low, 7–8 = medium and 15 = high. Reference standards were provided and served in 30 mL cups (Solo Cups). Panel evaluation was performed in duplicate, on two different days. All taste panels took place in isolated booths equipped with positive air pressure and under red lighting. Descriptors included orange, grapefruit, fruity-non-citrus, orange peel, green, stale, oxidized and typical HLB flavors; sweet, sour, umami, bitter and metallic tastes; and body, tingling, astringent and burning mouthfeel.

## 3. Results and Discussion

Orange juice was spiked with taste eliciting compounds, or others of similar structure, among which was sucrose, the major sugar in orange juice (imparts sweetness); citric acid, the major acid in orange juice (imparts sourness); potassium chloride, the major salt in orange juice (imparts saltiness or umami); limonin and nomilin (impart bitterness and/or metallic flavor); limonin glucoside (tasteless glycosylated form of limonin); hesperidin the major flavonoid in orange juice (contributes to astringency); and hesperitin, the aglycone form of hesperidin [22]. Table 1 shows the amount already present in the base juice. Generally sucrose concentrations decrease in HLB-affected orange juice whereas the increases often occur in the concentrations of citric acid, limonin, and nomilin, thereby disturbing the balance of flavors, causing sour and bitter off flavors. While the concentrations of potassium chloride is sometimes altered [23] by the disease, HLB-affected juice often has a salty or umami taste and the concentrations of hesperidin generally tend to increase along with other flavonoids in diseased fruit and juice. Although hesperidin is not associated with any taste stimuli, it may contribute to astringency, which is also enhanced in HLB juice [4,24]. These compounds are also important in orange as they contribute health benefits [25,26,27]. As for limonin glucoside, its importance lies in the fact that with fruit maturity, the bitter compound, limonin, is glycosylated to form the tasteless limonin glucoside. Although hesperetin is not a major secondary metabolite in orange, it was chosen due to its structural similarity with hesperidin, being its aglycone, which may give clues as to how molecular structure affects etongue classification of samples. All the replicates for both sensors showed similar trends, therefore, results from only one replication are shown, that was nonetheless, run on the etongue seven times, of which the last four runs are shown.

**Table 1:** Average concentrations and standard deviations of the compounds in base orange juice.

Compound	Average Concentration	Standard Deviation
	g/100 mL juice	
Sucrose	4	0.1
Citric Acid	1.2	0.2
Potassium	0.18	0.04
	µg/mL juice	
Nomilin	0.2	0.1
Limonin	1.7	0.4
Limonin Glucoside	155.4	5.9
Hesperidin	268.8	27.2

### 3.1. E-Tongue Analysis of Orange Juice Spiked with Sugars, Acids and Salt

**Figure 1:**
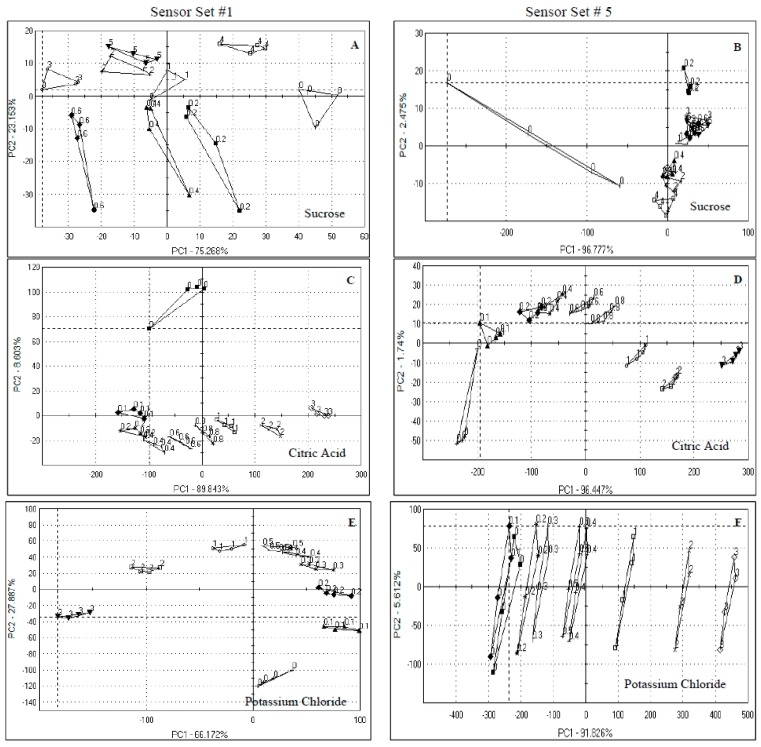
Principal component analysis of orange juice spiked with sucrose, citric acid and potassium chloride. Numbers in the graphs indicate the concentrations used (g/100 mL juice). Samples presented were analyzed by sensor set #1 and 5. (**A**) Sucrose by Set #1; (**B**) Sucrose by Set #5; (**C**) Citric acid by Set #1; (**D**) Citric acid by Set #5; (**E**) Potassium chloride by Set #1; (**F**) Potassium chloride by Set #5.

Sucrose is the major sugar in orange juice [4] and contributes along with glucose and fructose to its sweet taste [2] Since it was not possible to remove sugars from the base juice, experiments resulted in added sugars (or other chemicals) due to spiking with increasing amounts of sucrose followed by analysis on sensor set # 1 and #5 as shown in the PCA charts in Figure 1A,B, respectively.

For both sensor sets, the first two principal components (PCs) explained more than 98% of the data variations, with PC1 accounting for 75.3% and 96.8% for sensor set #1 and 5, respectively. However, there was more sample discrimination by sensor set #1 than by set #5, where the only differentiation was that of the unspiked sample (0) from the rest of the samples, resulting in larger Euclidean differences for sensor set #5 than for sensor set #1 (Table 2). The larger the Euclidean distance between two groups, the more different the samples are from each other and, therefore, the higher the discrimination. So, even though the PC1 explained more data variability for sensor set #5, it was not useful for discriminating among different spiked levels of sucrose. For sensor set #1, there was an increasing Euclidean distance between the unspiked sample (0) and spiked samples up to 0.6 g/100 mL level (Table 2) but higher concentrations (2–4 g/100 mL) were not well discriminated. This suggests that sensor set #1 was sensitive to discriminating samples spiked up to only 0.6 g/100 mL sucrose and anything above this level perhaps oversaturated the sensors, and therefore, no logical discrimination was observed. This can be explained in light of the high amount of sucrose (4.0 ± 0.1 g/100 mL, see Table 1) already present in the orange juice used as the base, which means adding more than 0.6 g/100 mL sucrose further increased the sucrose concentration, and therefore, reducing the sensor sensitivity, *i.e.*, over 6 g/100 mL sucrose in the juice (base juice = 4.0 g/100 mL + spiked 2.0 g/100 mL). In addition, it was noticed that the four sampling points per juice sample often introduced error picked up by PC2 or both PCs.

**Table 2:** Euclidean distances between unspiked base juice and spiked samples analyzed by electronic tongue sensor set (SS) #1/#5.

**Sucrose**			**Citric Acid**			**Potassium Chloride**		
**Base Juice**	**Spiked Juices**	**Distances SS#1/#5**	**Base Juice**	**Spiked Juices**	**Distances SS#1/#5**	**Base Juice**	**Spiked Juices**	**Distances SS#1/#5**
0	0.2	35/176	0	0.1	134/68	0	0.1	101/86
0	0.4	49/152	0	0.2	143/135	0	0.2	127/124
0	0.6	73/180	0	0.4	131/171	0	0.3	147/169
0	1	45/170	0	0.6	117/226	0	0.4	157/254
0	2	58/154	0	0.8	117/264	0	0.5	162/232
0	3	78/185	0	1	131/321	0	1	169/380
0	4	26/146	0	2	196/382	0	2	182/549
0	5	56/186	0	3	272/494	0	3	204/684
**Nomilin**			**Limonin**			**Limonin Glucoside**		
**Group 1**	**Group 2**	**Distances**	**Group 1**	**Group 2**	**Distances**	**Group 1**	**Group 2**	**Distances**
0	1	37/43	0	1	66/33	0	30	135/18
0	5	44/53	0	5	75/73	0	50	198/29
0	10	45/51	0	10	71/51	0	100	229/21
0	30	53/29	0	30	94/55	0	150	242/39
-	-	-	-	-	-	0	200	253/46
**Hesperidin**			**Hesperetin**					
**Base Juice**	**Spiked Juices**	**Distances SS#1/#5**		**Spiked Juices**	**Distances SS#1/#5**			
0	30	214/12	0	30	21/7			
0	50	253/15	0	50	25/13			
0	100	277/13	0	100	36/26			
0	200	288/12	0	200	53/36			
0	400	293/15	0	400	46/52			

Citric acid is the major acid in orange juice [4], and is responsible for the sour, tangy flavor. The PCA plots for samples spiked with different levels of citric acid for both sensor sets showed discrimination between the spiked samples with the first two PCs (Figure 1C,D) representing more than 98% of data variability and PC1 explaining most of the data variation (89.8% and 96.4% for sensor set #1 and #5, respectively).

Like the sucrose samples, PC1 of sensor set #5 (Figure 1D) had a higher contribution than that of sensor set #1 (Figure 1C), but the discriminating power of the sensor set #1 was higher for the lower levels of citric acid than that of sensor set #5 as shown by the Euclidean distances (see Table 2), but that is reversed for the higher levels. Also, it can be seen from Figure 1C,D that the samples were mainly separated by PC1 in set #5, while they were separated by PC1 and PC2 in set #1. This is significant as only 8.6% of the data is explained by PC2 in sensor set #5, which means that for citric acid, sensor set #5 performed somewhat better than did set #1 in differentiating the spiked samples from the unspiked sample for the concentrations tested. Based on the argument presented for sucrose, that perhaps the higher amounts of sucrose led to sensor saturation, it seems that sensor saturation was not reached with citric acid at 4.2 g/100 mL (1.2 g/100 mL + the highest spiked amount of 3.0 g/100 mL). As such, higher sensitivity of an e-tongue system to acids, salty and umami taste over sweet and bitter tastes have been reported [10], corroborating the better results obtained for citric acid spiked samples in this study.

Potassium chloride is the major salt in orange juice. The PCA plot shows that 94% of the variation was explained in the first two PCs with 27.9% explained by PC2 and 66.2% explained by PC1 for set #1 (Figure 1E) and 97.4% of the data variation using set #5 (Figure 1F) with most of the discrimination being in PC1 (91.8%). The differences in potassium chloride concentrations was picked up by both sensor sets, with the Euclidean distances being generally greater for set #5.

**Figure 2:**
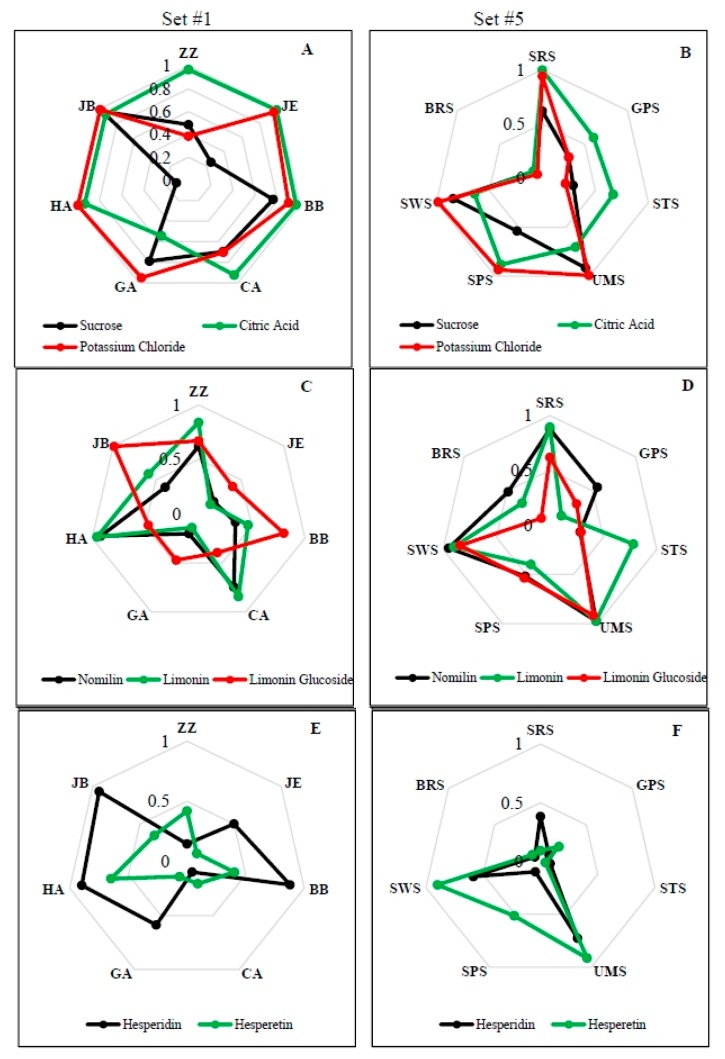
Radar plots showing discrimination power of the seven electronic tongue sensors, set #1 (**A**,**C**,**E**) and set #5 (**B**,**D**,**F**), for sucrose, citric acid, potassium chloride and nomilin, limonin, limonin glucoside, hesperidin, hesperetin.

Performance of sensors can be measured by discrimination power as shown by radar plots for both sensors sets used (Figure 2A,B). In set #1, six out of the seven sensors had discrimination powers close to maximum, *i.e.*, 1 for citric acid (on a scale of 0–1, Figure 2), whereas for potassium chloride spiked samples, five sensors were close to maximum, and for orange juice spiked with sucrose, only 1 sensor was close to maximum. Exceptions were sensors GA for citric acid and sensor CA and ZZ for potassium chloride. The situation was similar for sensor set #5, although only two and four sensors had discrimination power close to 1 for citric acid and potassium chloride spiked juices, respectively. Interestingly, the SWS sensor, which is claimed by the manufacturer to respond to sweetness, responded well to sucrose as well as potassium chloride, and the sourness sensor, SRS, responded strongly to citric acid and potassium chloride.

### 3.2. E-Tongue Analysis of Orange Juice Spiked with Limonoids

**Figure 3:**
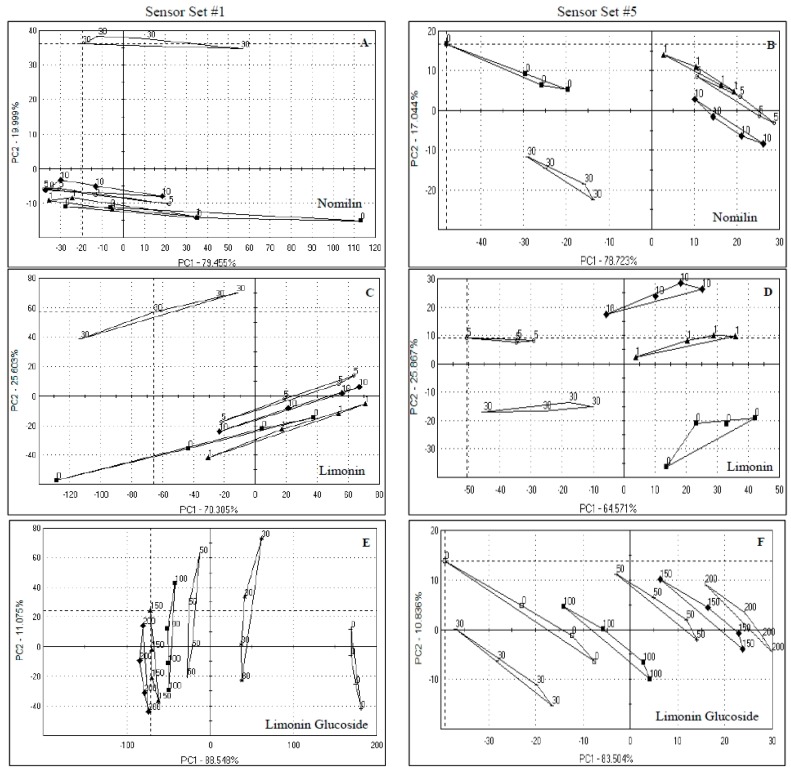
Principal component analysis of orange juice spiked with nomilin, limonin and limonin glucoside. Numbers in the graphs indicate the concentrations used (µg/mL juice). Samples presented were analyzed by sensor set #1 and 5. (**A**) Nomilin by Set #1; (**B**) Nomilin by Set #5; (**C**) Limonin by Set #1; (**D**) Limonin by Set #5; (**E**) Limonin glucoside by Set #1; (**F**) Limonin glucoside by Set #5.

Nomilin is present in lower concentrations than limonin in orange juice and imparts a bitter and metallic taste [22]. For sensor set #1 PCA plot (Figure 3A), 99.4% of the data was explained in the first two PCs, mostly by PC1 (79.5%), but the different concentrations were not well separated, especially within the physiologically relevant range in which this compound is found in orange fruit and juice. The separation of nomilin levels was much better using sensor set #5 (Figure 3B) where 95.8% of the variation was explained in the first 2 PCs, with most of it in PC1 (78.7%). The Euclidian distances were generally greater for sensor set #5 than for sensor set #1 for nomilin (Table 2).

The situation was similar for limonin (Figure 3C), the more abundant bitter limonoid in orange juice, where 95.9% of the data was explained, again mostly on PC1 (70.3%) for sensor set #1, with no meaningful separation of the more relevant lower levels. As with nomilin, sensor set #5 did a much better job in separating the spiked levels of limonin (Figure 3D), mostly on PC1, but with some of the variation explained also by PC2 (90.5% total for limonin with 64.6% on PC1), however, Euclidean distances were higher for sensor set #1 (Table 2), although the discrimination was not useful being mainly between the highest spiked level and the rest of the samples.

For limonin glucoside, sensor set #1 (Figure 3E) discriminated quite well explaining 99.7% of the variation, mostly on PC1 (88.5%) and for set #5 (Figure 3F), 94.3% of the variation was explained, again mostly by PC1 (83.5%), with sensor set #1 having much higher Euclidean distances (Table 2). Limonin glucoside is the glycosylated form of limonin, and is non bitter or tasteless. This glycosylation occurs as the orange fruit mature on the tree [28], thus fruit harvested earlier in the season tend to be perceived as more bitter than those harvested later in the season [29,30].

In terms of sensor performance, responses to both nomilin and limonin were similar, whereas different sensors were more active for limonin glucoside for both sensor sets (Figure 2C,D). In set #1, sensors HA, CA and ZZ showed the most response to nomilin and limonin, but exhibited a lesser response to limonin glucoside, and the opposite was true for sensors JB and BB. In set # 5, sensors SWS, SRS and UMS had discrimination power close to 1 for all the compounds. Differences were found for BRS, GPS and STS where BRS and GPS had higher responses to nomilin and STS to limonin. Limonin glucoside had the lowest discrimination index for these sensors (BRS, GPS and STS). Interestingly, the BRS sensor, which is thought to respond to bitter taste, did not respond well to either nomilin or limonin, the two bitter compounds in HLB-affected orange juice.

### 3.3. E-Tongue Analysis of Orange Juice Spiked with Flavonoids

**Figure 4:**
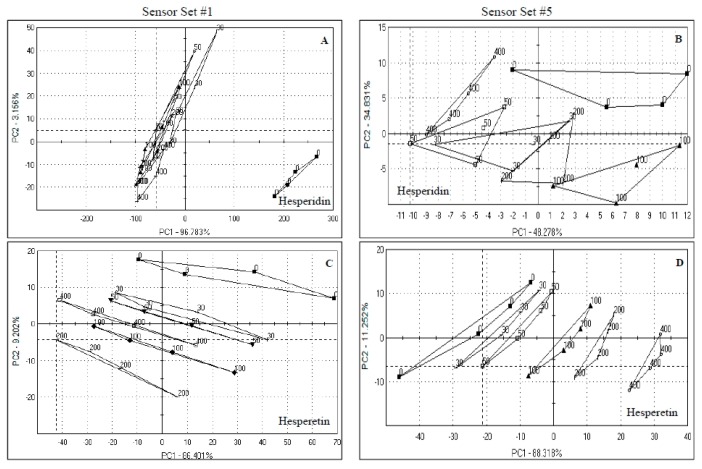
Principal component analysis of orange juice spiked with hesperidin and hesperetin. Numbers in the graphs indicate the concentrations used (µg/mL juice). Samples presented were analyzed by sensor set #1 and 5. (**A**) Hesperidin by Set #1; (**B**) Hesperidin by Set #5; (**C**) Hesperetin by Set #1; (**D**) Hesperetin by Set #5.

Flavonoids can contribute astringency to orange fruit and juice [2]. The PCA plot shows that spiking levels for hesperidin were not well discriminated by sensor set #1 or #5 (Figure 4). For sensor set #1 (Figure 4A), 100% of the data was explained, mostly on PC1 (96.8%), but only between the unspiked control (0) and the spiked samples. Sensor set #5 (Figure 4B) did a little better with 83.1% of the variation was explained by both PCs (48.3% and 34.8% on PC1 and PC2, respectively). The Euclidean distances were much higher for sensor set #1, but only because of the large distance between the unspiked control and the rest of the samples (Table 2).

Hesperetin is the aglycone form of hesperidin, and present in orange juice only at very low levels [4]. There was fairly good separation for this compound by sensor set #1 (Figure 4C) and even better by sensor set #5 (Figure 4D). For sensor set #1, 95.6% of the data was explained in 2 PCs, but mostly by PC1 (86.4%), whereas for sensor set #5, 99.5% of the variation was explained on 2 PCs, again mostly on PC 1 (88.3%). The Euclidean distances were slightly higher for sensor set #1 than for sensor set #5 (Table 2).

Overall, the radar plots for both hesperidin and hesperetin (Figure 2E,F) showed that both sensor sets did not have much of a response for most of the sensors compared to juice spiked with other chemicals. This explains the relatively poor segregation observed in the PCA diagrams (Figure 4A–D). Sensor set #1 had a better response to hesperidin whereas set #5 had a better response to hesperetin.

### 3.4. E-Tongue Analysis of Orange Juices Affected by Harvest Maturity and HLB Disease

Since sensor set #5 was found in this study to have better discrimination for most of the spiked compounds than sensor set #1, analysis of field oranges was conducted using sensor set #5 only. Hamlin oranges, one of the major juice oranges in Florida [31], was harvested weekly from December to March in 2011–2012 in southern Florida and the juice processed and pasteurized in a commercial pilot plant and analyzed by the e-tongue.

**Figure 5:**
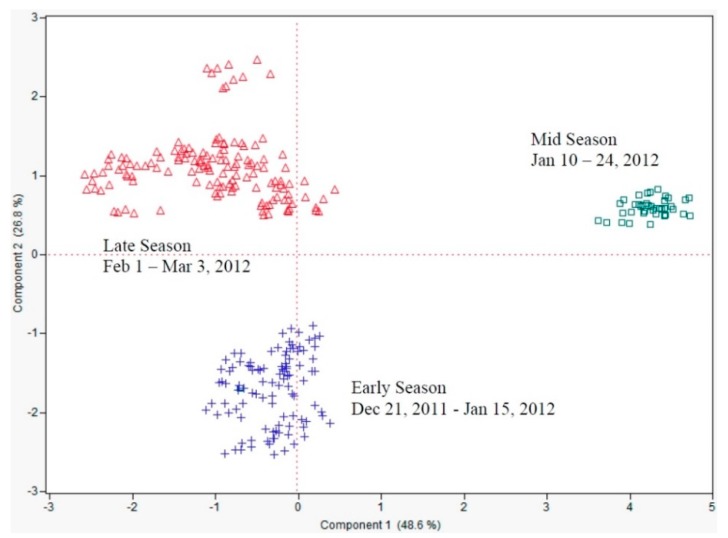
Principal components analysis showing e-tongue separation (sensor set #5) of Hamlin orange juice from fruit harvested over the season (early, mid and late harvests) for the 2011–2012 season in South Florida.

The results showed excellent separation of early season (21 December 2011 to 5 January 2012), mid-season (10 January 2012 to 24 January 2012) and late season (1 February 2012 to 3 March 2012) fruit juice (Figure 5) on the first two PCs explaining 75.4% of the data, mostly on PC1 (48.6%).

**Figure 6:**
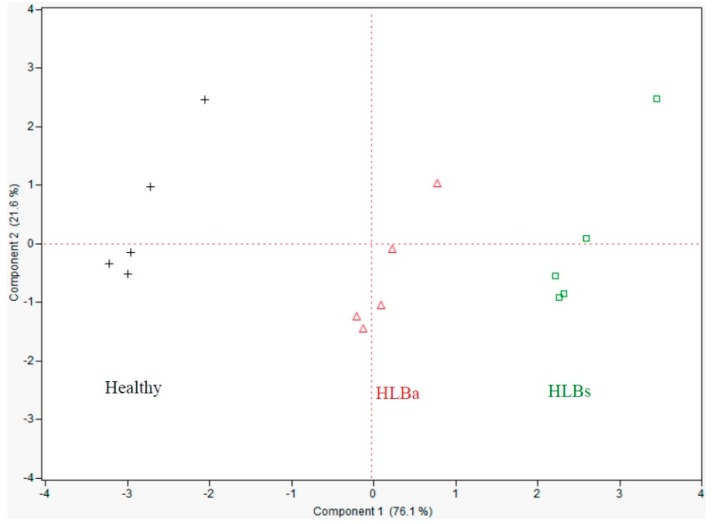
Principal components analysis showing e-tongue separation (sensor set #5) of Hamlin orange juice from fruit harvested from healthy, HLB asymptomatic (HLBa) and HLB symptomatic (HLBs) trees in February 2011 in South Florida.

Juice from fruit harvested from healthy and HLB-affected trees from the same groves, for which the latter were separated into HLBs and HLBa fruit before juicing, discriminated for disease state (healthy *versus* HLB) and disease severity (HLBs *versus* HLBa) (Figure 6) on the first two PCs explaining 97% of the data on two PCs, mostly on PC1 (76%).

**Figure 7:**
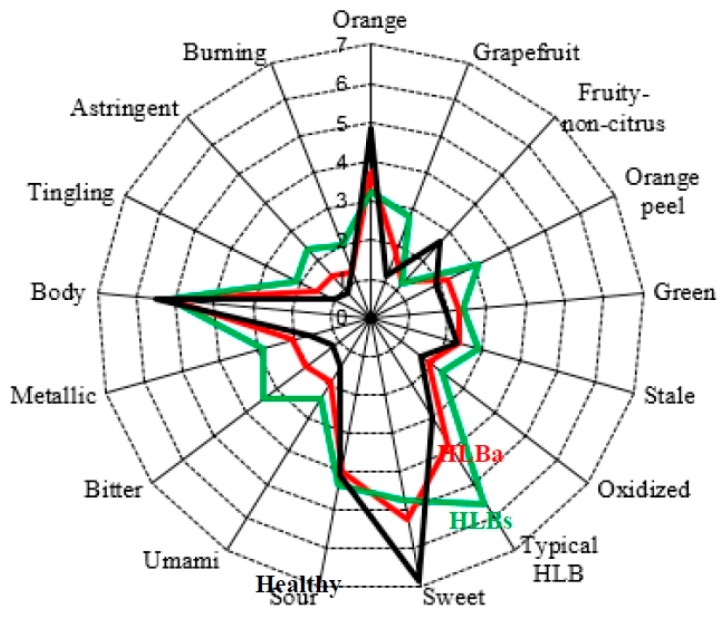
Trained descriptive sensory analysis of fruit from healthy (Healthy) or HLB affected trees were separated into those that were symptomatic (HLBs) or asymptomatic (HLBa) for the disease prior to juicing (HLB = Huanglongbing disease).

Some of the healthy, HLBs and HLBa fruit were analyzed by a sensory panel (Figure 7), showing differences for many descriptors with healthy fruit juice having more orange and fruity-non-citrus flavors, as well as sweet taste than the juice from HLBs fruit. The fruit juice from HLBs fruit had more grapefruit, peel, stale and typical HLB flavors; umami, bitter and metallic tastes; tingling, astringent and burning mouthfeel. The juice made from HLBa fruit was generally between the healthy and HLBs fruit juice ratings for these descriptors, agreeing with the e-tongue analysis. The sweet taste difference was likely due to the sugars in the juice [2], the bitterness to the bitter limonoids [32] and the astringency due to the flavonoids [24].

## 4. Conclusions

For the spiked compounds, sucrose seemed to overload overwhelm the sensors at the higher levels for both sensor sets #1 and #5 while citric acid and potassium chloride were quite well separated by both sensor sets. The aglycones, nomilin and limonin, were not separated well at the lower, more relevant ranges, (<10 µL/mL) by sensor #1, whereas the glycosylated form of limonin, limonin glucoside, was. In contrast, nomilin, limonin and limonin glucoside were all quite well separated by sensor set #5. Meanwhile the aglycone, hesperitin, was better discriminated than the glycosylated hesperidin by sensor set #1 and #5, so the presence or absence of the sugar moiety, did not consistently affect the ability of the etongue sensors to discriminate for different compounds. Orange juice samples harvested from different seasons and with different HLB disease severity were also well discriminated by the electronic tongue system used in this study, suggesting it to be a useful tool for quality determination in orange juice and other beverages.

## Note

This article is a US Government work and is in the public domain in the USA. Mention of a trademark or proprietary product is for identification only and does not imply a guarantee or warranty of the product by the US Department of Agriculture. The US Department of Agriculture prohibits discrimination in all its programs and activities on the basis of race, color, national origin, gender, religion, age, disability, political beliefs, sexual orientation, and marital or family status.

## References

[1] FDOC Florida Citrus Outlook 2014–15 Season. https://fdocgrower.app.box.com/shared/2n5zfo2cur/1/76190650/22178980651/1.

[2] Plotto A., Baldwin E., McCollum G., Manthey J., Narciso J., Irey M. (2010). Effect of Liberibacter infection (Huanglongbing or “Greening” Disease) of citrus on orange juice flavor quality by sensory evaluation. J. Food Sci..

[3] Raithore S., Dea S., Plotto A., Bai J., Manthey J., Narciso J., Irey M., Baldwin E. (2015). Effect of blending Huanglongbing (HLB) disease affected orange juice with juice from healthy orange on flavor quality. LWT Food Sci. Technol..

[4] Baldwin E., Plotto A., Manthey J., McCollum G., Bai J., Irey M., Cameron R., Luzio G. (2010). Effect of Liberibacter infection (Huanglongbing disease) of citrus on orange fruit physiology and fruit/fruit juice quality: Chemical and physical analyses. J. Agric. Food Chem..

[5] Dagulo L., Danyluk M.D., Spann T.M., Valim M.F., Goodrich-Schneider R., Sims C., Rouseff R. (2010). Chemical characterization of orange juice from trees infected with citrus greening (Huanglongbing). J. Food Sci..

[6] Plotto A. Effect of nutritional treatments on Huanlongbing infected citrus flavor quality by sensory evaluation.

[7] Legin A., Rudnitskaya A., Lvova L., Vlasov Y., Di Natale C., D’Amico A. (2003). Evaluation of Italian wine by the electronic tongue: Recognition, quantitative analysis and correlation with human sensory perception. Anal. Chim. Acta.

[8] Rudnitskaya A.P.E., Kirsanov D., Lammertyn J., Nicolai B., Saison D., Delvaux F.R., Delvaus F., Legin A. (2009). Instrumental measurement of beer taste attribues using an electronic tongue. Anal. Chim. Acta.

[9] Beullens K., Kirsanov D., Irudayaraj J., Rudnitskaya A., Legin A., Nicolaï B.M., Lammertyn J. (2006). The electronic tongue and atr–ftir for rapid detection of sugars and acids in tomatoes. Sens. Actuators B Chem..

[10] Dias L.A., Peres A.M., Veloso A.C.A., Reis F.S., Vilas-Boas M., Machado A.A.S.C. (2009). An electronic tongue taste evaluation: Identification of goat milk adulteration with bovine milk. Sens. Actuators B Chem..

[11] Liu M.W.J., Li D., Wang M. (2012). Electronic tongue coupled with physicochemical analysis for the recognition of orange beverages. J. Food Qual..

[12] Lindemann B. (2001). Receptors and transduction in taste. Nature.

[13] Vlasov Y.L.A., Rudnitskaya R., Natale C.D., D’Amico A. (2005). Nonspecific sensor arrays (“Electronic tongue”) for chemical analysis of liquids. Pure Appl. Chem..

[14] Legin A., Rudnitskaya A., Vlasov Y., Di Natale C., Davide F., D’Amico A. (1997). Tasting of beverages using an electronic tongue. Sens. Actuators B Chem..

[15] Gutiérrez J.M., Haddi Z., Amari A., Bouchikhi B., Mimendia A., Cetó X., del Valle M. (2013). Hybrid electronic tongue based on multisensor data fusion for discrimination of beers. Sens. Actuators B Chem..

[16] Bueno L., de Araujo W., Salles M., Kussuda M., Paixão T. (2014). Voltammetric electronic tongue for discrimination of milk adulterated with urea, formaldehyde and melamine. Chemosensors.

[17] Ciosek P.M.R., Dybko A., Wroblewski W. (2007). Potentiometric electronic tongue based on integrated array of microelectrodes. Sens. Actuators B Chem..

[18] Ding F.L.B., Deng X., Wang Z., Xie Z., Fang Y., Xu J. (2010). Delayed bitterness of six sweet oranges (*Citrus sinensis* osbeck). J. Huazhong Agric. Univ..

[19] Baldwin E.A., Bai J., Plotto A., Dea S. (2011). Electronic noses and tongues: Applications for the food and pharmaceutical industries. Sensors.

[20] Jagoueix S., Bove J.M., Garnier M. (1996). Pcr detection of the two “*Candidatus*” *Liberibacter* species associated with greening disease of citrus. Mol. Cell. Probes.

[21] Bai J., Baldwin E., Liao H.L., Zhao W., Kostenyuk I., Burns J., Irey M. (2013). Extraction of DNA from orange juice, and detection of bacterium *Candidatus* Liberibacter asiaticus by real-time PCR. J. Agric. Food Chem..

[22] Dea S., Plotto A., Manthey J.A., Raithore S., Irey M., Baldwin E. (2013). Interactions and thresholds of limonin and nomilin in bitterness perception in orange juice and other matrices. J. Sens. Stud..

[23] Baldwin E., Bai J., Plotto A., Manthey J., Narciso J., Dea S., Irey M. (2012). Effect of nutritional spray regimes on orange juice flavor quality and juice Liberibacter (*C*Las) DNA detection. Proc. Fla. State Hortic. Soc..

[24] Havekotte M., Hofmann T., Rakofsky T., Nagle C., Morello M., Jordan R. (2012). Control of Flavor Characteristics of Fruit Juice. European Patent.

[25] Grosso G., Galvano F., Mistretta A., Marventano S., Nolfo F., Calabrese G., Buscemi S., Drago F., Veronesi U., Scuderi A. (2013). Red orange: Experimental models and epidemiological evidence of its benefits on human health. Oxidative Med. Cell. Longev..

[26] Ejaz S., Ejaz A., Matsuda K., Lim C.W. (2006). Limonoids as cancer chemopreventive agents. J. Sci. Food Agric..

[27] Yu J., Wang L., Walzem R.L., Miller E.G., Pike L.M., Patil B.S. (2005). Antioxidant activity of citrus limonoids, flavonoids, and coumarins. J. Agric. Food Chem..

[28] Endo T.K.M., Shimada T., Moriguchi T., Hidaka T., Matsumoto R., Hasegawa S., Omura M. (2002). Modification of limonoid metabolism in suspension cell culture of citrus. Plant Biotechnol..

[29] Jungsakulrujirek S., Noomhorm A. (1998). Effect of harvesting time and fruit size on titratable acidity, soluble solid and distribution of limonin in thai tangerine juice. Int. J. Food Sci. Technol..

[30] Bai J., Baldwin E., Plotto A., Manthey J.A., McCollum G., Irey M. (2009). Influence of harvest time on quality of “Valencia” oranges and juice. Proc. Fla. State Hortic. Soc..

[31] Hodgson R.W., Reuther W., Webber H.J., Batchelor L.D. (1967). Horticultural varieties of citrus. The Citrus Industry.

[32] Hasegawa S., Bennett R.D., Verdon C.P. (1980). Limonoids in citrus seeds: Origin and relative concentration. J. Agric. Food Chem..

